# Effect of Fruit Juice on Glucose Control and Insulin Sensitivity in Adults: A Meta-Analysis of 12 Randomized Controlled Trials

**DOI:** 10.1371/journal.pone.0095323

**Published:** 2014-04-17

**Authors:** Bin Wang, Kai Liu, Mantian Mi, Jian Wang

**Affiliations:** 1 Research Center for Nutrition and Food Safety, Institute of Military Preventive Medicine, Third Military Medical University, Chongqing Key Laboratory of Nutrition and Food Safety, Chongqing Medical Nutrition Research Center, Chongqing, P. R. China; 2 Department of Nutrition, Xinqiao Hospital, Third Military Medical University, Chongqing, P. R. China; University of Chieti, Italy

## Abstract

**Background:**

Diabetes mellitus has become a worldwide health problem. Whether fruit juice is beneficial in glycemic control is still inconclusive. This study aimed to synthesize evidence from randomized controlled trials on fruit juice in relationship to glucose control and insulin sensitivity.

**Methods:**

A strategic literature search of PubMed, EMBASE, and the Cochrane Library (updated to March, 2014) was performed to retrieve the randomized controlled trials that evaluated the effects of fruit juice on glucose control and insulin sensitivity. Study quality was assessed using the Jadad scale. Weighted mean differences were calculated for net changes in the levels of fasting glucose, fasting insulin, hemoglobin A1c (HbA1c), and homeostatic model assessment of insulin resistance (HOMA-IR) using fixed- or random-effects model. Prespecified subgroup and sensitivity analyses were performed to explore the potential heterogeneity.

**Results:**

Twelve trials comprising a total of 412 subjects were included in the current meta-analysis. The numbers of these studies that reported the data on fasting glucose, fasting insulin, HbA1c and HOMA-IR were 12, 5, 3 and 3, respectively. Fruit juice consumption did not show a significant effect on fasting glucose and insulin concentrations. The net change was 0.79 mg/dL (95% CI: −1.44, 3.02 mg/dL; *P* = 0.49) for fasting glucose concentrations and −0.74 µIU/ml (95% CI: −2.62, 1.14 µIU/ml; *P* = 0.44) for fasting insulin concentrations in the fixed-effects model. Subgroup analyses further suggested that the effect of fruit juice on fasting glucose concentrations was not influenced by population region, baseline glucose concentration, duration, type of fruit juice, glycemic index of fruit juice, fruit juice nutrient constitution, total polyphenols dose and Jadad score.

**Conclusion:**

This meta-analysis showed that fruit juice may have no overall effect on fasting glucose and insulin concentrations. More RCTs are warranted to further clarify the association between fruit juice and glycemic control.

## Introduction

Diabetes mellitus is now one of the most challenging health problems globally. As reported by the International Diabetes Federation (IDF), more than 371 million people worldwide have diabetes in 2012, and this number is projected to increase to 552 million people by 2030 if no urgent action is taken [1], [2]. In addition, numerous people with impaired glucose tolerance (IGT) or impaired fasting glycaemia (IFG) are at high risk of progressing to type 2 diabetes mellitus (T2DM) [3], [4]. It has been proven that T2DM and its complications are the major cause of disability, reduced quality of life and premature death, imposing a heavy burden on patients and society [5]. Therefore, the importance of efforts to reduce the incidence of diabetes has never been greater.

Accumulating evidence suggests that lifestyle changes, including eating healthy foods can help prevent or delay the development of T2DM [6]–[8]. Fruits are rich in fiber, antioxidants, and phytochemicals that may have beneficial effects on health, and thus are recommended for the primary prevention of T2DM [9]. A recent study also suggested that the consumption of specific whole fruits is related to a significant reduction of T2DM risk [10]. In contrast, evidence on whether fruit juices possess similar protective effects attribute to the whole fruits is still inconclusive [11]–[13]. According to the recommendation by 2010 Dietary Guideline for Americans, fruit juice is considered to be less desirable because it has less dietary fiber than whole fruit [14]. Fruit juice is also criticized by its concentrated or additionally supplemented sugars and contributing the extra calories when consumed in excess [15]. However, Schulze et al. [16] found that fruit fiber was not significantly related to the lower risk of diabetes based on the data of previous prospective studies [17]–[23]. Additionally, it has been demonstrated that although fruit juice is deficient in fiber, other important preventive nutritional components, such as antioxidants and phytochemicals (e.g. polyphenols), are present in fruit juice [24]. In view of these dual properties of fruit juice, great concern has been aroused to identify the effect of fruit juice on T2DM risk. To date, several RCT studies have been conducted to evaluate the association between fruit juicy consumption and glycemic control, but the results have been conflicting. Therefore, we conducted this meta-analysis to synthesize evidence from previous RCTs and provide a more precise estimate of the effect of fruit juice on glucose control and insulin sensitivity based on the PRISMA guidelines.

## Methods

### Search Strategy

PubMed (updated to March 2014; http://www.ncbi.nlm.nih.gov/pubmed/), Embase (1980 to March 2014; http://www.embase.com/), the Cochrane Library (1985 to March 2014; http://www.cochrane.org/) database, and reference lists and reviews were searched for RCTs designed to assess the effects of fruit juice on glucose control and insulin sensitivity in humans. The structured search strategies used the text words juice or juices limited in abstract/title. The search was restricted to the reports of RCTs in humans (e.g., the search strategy for Embase is: juice:ab,ti OR juices:ab,ti AND [randomized controlled trial]/lim AND [humans]/lim AND [embase]/lim AND [1980–2014]/py).

### Study Selection

Studies were selected for this analysis if they 1) were RCTs conducted in human subjects; 2) used a concurrent control group (such as placebo beverage, water, or controlled drink) for the fruit juice treatment group and the difference between the control and treatment group was fruit juice. 3) included subjects ingesting fruit juice for ≥2 wk (to remove acute or very short-term studies); 4) provided the information of baseline and endpoint values or the difference of fasting glucose and insulin concentrations with SD or SEM or 95% CI (when necessary, the authors were contacted to obtain the unavailable data); 5) did not give fruit juice as part of a multi-component supplement.

### Quality Assessment

The methodological quality of all studies was evaluated using the following criteria: 1) randomization; 2) double blinding; 3) withdrawals (number and reasons); 4) allocation concealment; and 5) generation of random numbers. One point was given for each area addressed in the study design and the total Jadad score ranges from a minimum of 0 to a maximum of 5 points [25]. The trials with a score of ≥4 were classified as high quality, whereas those receiving a score of <4 were considered as lower quality.

### Data Extraction

All data were screened by two investigators (BW and KL) independently with any disagreement resolved by consensus and then collected onto a pre-designed template that included the following items: 1) study characteristics including authors, publication year, sample size, study design, population information, study duration, total polyphenols dose, type of intervention and type of diet; 2) net changes in fasting glucose and insulin concentration, hemoglobin A1c (HbA1c) and the homeostatic model assessment of insulin resistance (HOMA-IR). All values were converted to mmol/L for glucose and µIU/ml for insulin by using conversion factors 1 mg/dL = 0.0556 mmol/L for glucose and 1 pmol/L = 6.945 µIU/ml for insulin concentrations. If primary outcome and secondary outcome concentrations were reported several times in different stages of trials, only values representing the final outcomes at the end of trials were extracted for our meta-analysis.

### Statistical Analysis

All statistical analysis in our meta-analysis was performed using STATA, version 11 (StataCorp, College Station, TX, USA). Treatment effects were defined as weighted mean difference (WMD) and 95% CIs in concentrations of fasting glucose and insulin, and values of HbA1c and HOMA-IR. The statistical heterogeneity was examined using Cochran’s test (a *P* value <0.1 was considered statistically significant) and *I* ^2^ tests (*I* ^2^>50%, significant heterogeneity) [26]. A random or fixed effects model was used for heterogeneous or non-heterogeneous data, respectively. Nonetheless, fixed effects model was used when less than 5 trials were included in the analysis due to uncertainty in the prediction of heterogeneity [27]. Funnel plots and the Egger’s tests were used to assess the potential publication bias when 10 or more studies were included in meta-analysis.

When not directly available, SD values were calculated from standard errors, 95% CIs, *P*-values, or t-values. In addition, we assumed a correlation coefficient of 0.5 between baseline and final values, as suggested by Follmann et al [28]. To assess the possible source of heterogeneity between the studies, subgroup analyses were conducted by comparing the study results of population region, baseline glucose concentration, study design, duration, type of fruit juice, glycemic index (GI) of fruit juice, fruit juice nutrient constitution, total polyphenols dose and Jadad score. GI values were obtained from the international GI database [29]. Additional sensitivity analyses were also performed in accordance with the Handbook for Systematic Review of Interventions of Cochrane software (Version 5.0.2; The Cochrane Collaboration, Oxford, UK).

## Results

### Results of Literature Search

**Figure 1:**
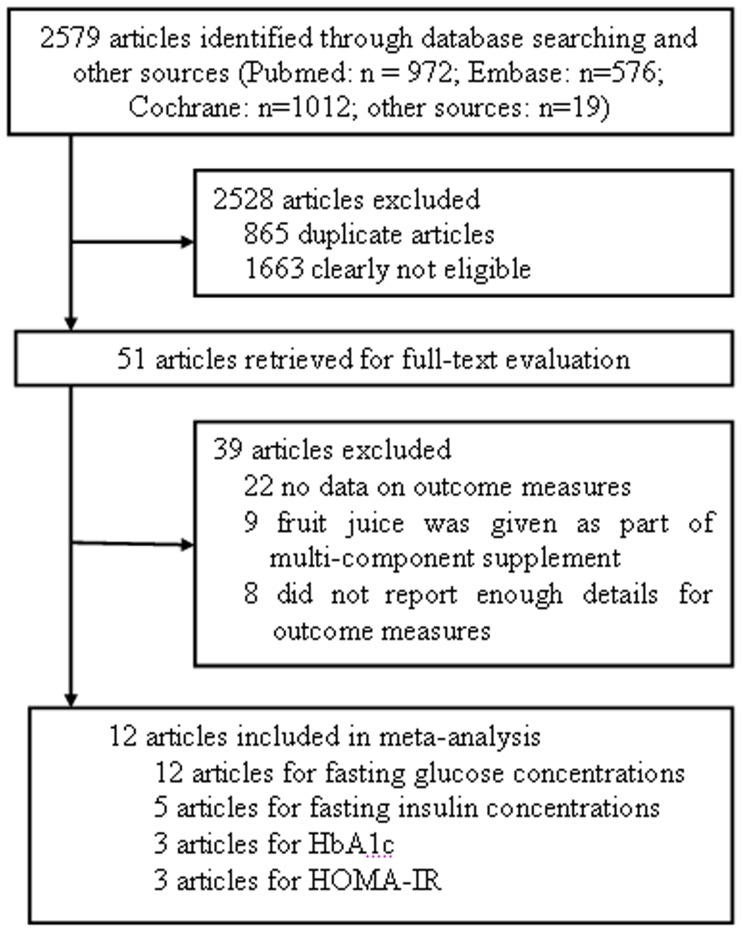
Flow diagram showing the number of citations retrieved by individual searches of articles included in the review. HbA1c, hemoglobin A1c; HOMA-IR, the homeostatic model assessment of insulin resistance.

Detailed steps of the literature search are shown in **Figure 1**. Of the 2579 initially identified reports, 2528 articles were excluded either because they were duplicate or not relevant to the current meta-analysis. Therefore, 51 potentially relevant articles were further examined. Of these, an additional 39 articles were excluded for the following reasons: 22 articles did not have the data on outcome measures, 9 articles treated the subjects with multi-component supplement, 8 articles did not report enough details of SD or baseline or endpoint or mean difference for primary or secondary outcome measures [30]–[37]. We contacted the main authors of these 8 studies by email, but only one replied and the requested data was no longer available [31]. Thus, 12 articles were finally selected for inclusion in the meta-analysis [38]–[49].

### Study Characteristics

The characteristics of trials included in the meta-analysis are shown in **Table 1** and **Table S1**. A total of 12 trials involved 412 subjects and the trials varied in size from 12 to 63 subjects. Total polyphenols content of fruit juice ranged from 341.9 to 2660 mg/d (median: 933.6 mg/d). The study duration varied from 4 wk to 3 mo (median: 7 wk). Seven of 12 RCTs included subjects with hyperglycemia (>110 mg/dL), while the remaining 5 studies selected participants with normal fasting glucose concentrations. Most of the studies (8 of 12) used parallel design. Of the 10 studies which maintained a usual diet, 7 studies suggested the participants avoid the intake of other dietary confounding factors such as wine, green tea or soy products (**Table 1**). In addition, 10 trials used fruit juice with low GI value (≤55) and the rest 2 studies used median GI fruit juice (56 to 69) [29]. Eight of the 12 included studies reported no significant changes in body weight, and the remaining 4 studies did not report the information of body weight. The participants in all included studies consumed the fruit juices and placebo drinks in the free-living situation. Only 2 studies used ellagic acid, or β-cryptoxanthin plus vitamin C as the biomarkers to evaluate the intervention compliance [45], [48], while 9 studies reported that they have assessed the compliance by periodical in-person interview, or the questionnaire survey, or counting the unconsumed drinks to record the consumption information, etc (**Table S1**).

**Table 1:** Characteristics of 12 randomized controlled trials included in analysis.

Study	No. ofsubjects	Country	Studydesign	Participants	Duration	Juice group (Totalpolyphenols; Energy)	Control group	Type of diet
Reshef 2005	12	Israel	Crossover	Stage I hypertension	5 wk	500 ml sweetie fruit juice(444.5 mg/d; NR)	Placebo (low-flavonoidsweetie juice: 115 mg/d)	Usual diet
Summer 2005	39	US	Parallel	Coronary heart disease	3 mo	240 ml pomegranate juice(NR; NR)	Placebo (modifiedsports beverage)	NR
Bannni 2006	23	US	Parallel	Type 2 diabetes mellitus	28 d	150 ml muscadine grapejuice (NR; NR)	No intervention	Usual diet
Cerda 2006	30	Spain	Parallel	Chronic obstructivepulmonary disease	5 wk	400 ml pomegranate juice(2660 mg/d; NR)	Placebo (synthetic orangeflavoured drink)	Controlled diet, limit berries, pomegranates,chocolate, nuts and wine
Hollis 2010	51	US	Parallel	Overweight	12 wk	480 ml concord grapejuice (933.6 mg/d; 350 kcal/d)	Placebo (grape-flavored drink)	Usual diet, avoid the intake of other juices
Basu 2010a	48	US	Parallel	Metabolic syndrome	8 wk	50 g freeze-dried blueberryjuice (1624 mg/d; 174 kcal/d)	Water (similar fluid intake)	Usual diet, avoid the intake of other berries,green tea, cocoa, and soy
Dohadwala 2010	63	US	Crossover	Stage I hypertension	8 wk	490 ml concord grape juice(965 mg/d; 327 kcal/d)	Placebo beverage	Usual diet, avoid the intake of grape juice,wine, grape products, green or black tea,dark juices
Basu 2010b	27	US	Parallel	Metabolic syndrome	8 wk	50 g freeze-dried strawberryjuice (2160 mg/d; 150 kcal/d)	Water (similar fluid intake)	Usual diet, avoid the intake of other berries
Gonzalez-Ortiz 2011	20	US	Parallel	Obesity	1 mo	120 ml pomegranate juice(NR; NR)	Placebo (NR)	Usual diet
Dohadwala 2011	44	US	Crossover	Coronary artery disease	4 wk	480 ml cranberry juice(835 mg/d; NR)	Placebo beverage(no polyphenols)	Usual diet, avoid the intake of grape juice,wine, grape products, green or black tea,dark juices
Morand 2011	24	France	Crossover	Overweight	4 wk	500 ml orange juice(341.9 mg/d; 194 kcal/d)	Control drink (only containsmatched sugar composition)	Usual diet, avoid the intake of citrus-containing foods and limit tea, coffee,cocoa, wine, fruit juice ≤200 mL/d
Basu 2011	31	US	Parallel	Metabolic syndrome	8 wk	240 ml low-energy cranberryjuice (458 mg/d; 167 kcal/d)	Placebo (polyphenol-free)	Usual diet, avoid the intake of berries,green tea, cocoa, and soy products

The studies by Reshef (2005), Cerda (2006), Hollis (2010), Dohadwala (2010), Dohadwala (2011), and Basu (2011) used fruit juice with polyphenols as main nutrients, and the studies by Summer (2005), Bannni (2006), Basu (2010a), Basu (2010b), Gonzalez-Ortiz (2011), and Morand (2011) used fruit juice with multi-nutrients (polyphenols, vitamins, and sugar et al.); a usual diet was similar to a conventional diet.

NR, not reported.

### Data Quality

Study qualities of the selected trials were assessed by the Jadad scale [25], and the results were diverse. Six trials were classified as high quality (Jadad score ≥4) [39], [41], [42], [44], [48], [49], and the remaining 6 trials were classified as low quality (Jadad score <4). All of the 6 high-quality trials had the clearly adequate allocation concealment (ie, allocated by a third party or used opaque envelopes), and 2 high-quality trials reported the generation of random numbers or randomization list. The details of dropouts were reported in 10 trials [38],[39],[41]–[45],[47]–[49].

### Effect of Fruit Juice on Glucose Control and Insulin Sensitivity

**Figure 2:**
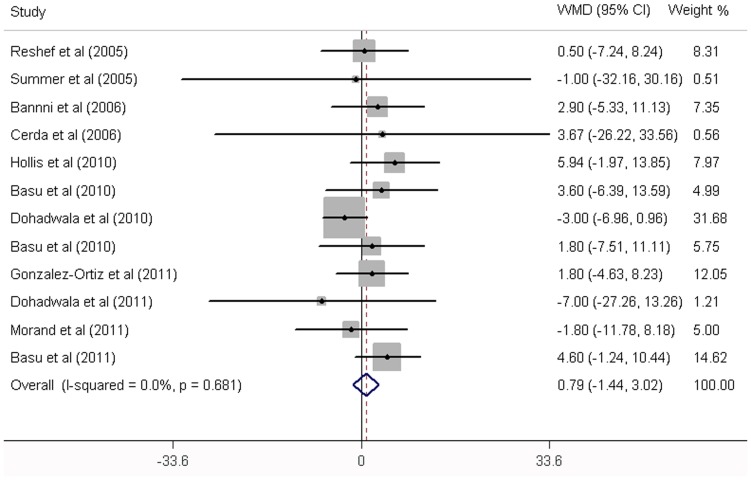
Meta-analysis of effects of fruit juice on fasting glucose concentrations. The result was obtained from a fixed-effects model. Sizes of data markers indicate the weight of each study in this analysis. WMD, weighted mean difference.

**Figure 3:**
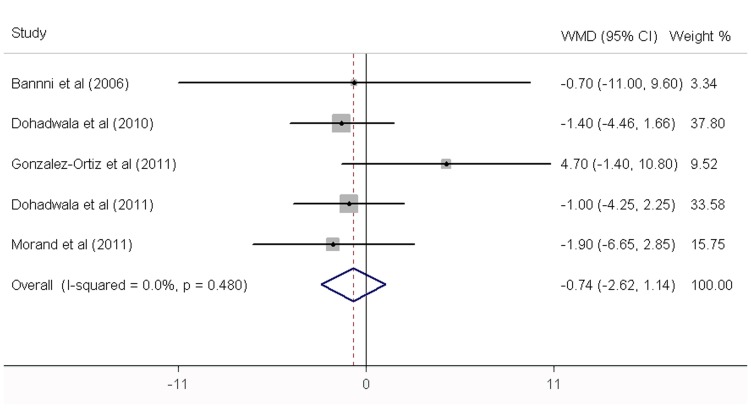
Meta-analysis of effects of fruit juice on fasting insulin concentrations. The result was obtained from a fixed-effects model. Sizes of data markers indicate the weight of each study in this analysis. WMD, weighted mean difference.

**Figure 4:**
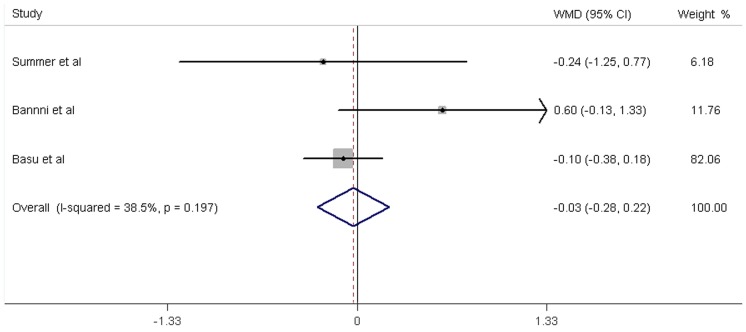
Meta-analysis of effects of fruit juice on HbA1c concentrations. The result was obtained from a fixed-effects model. Sizes of data markers indicate the weight of each study in this analysis. WMD, weighted mean difference.

**Figure 5:**
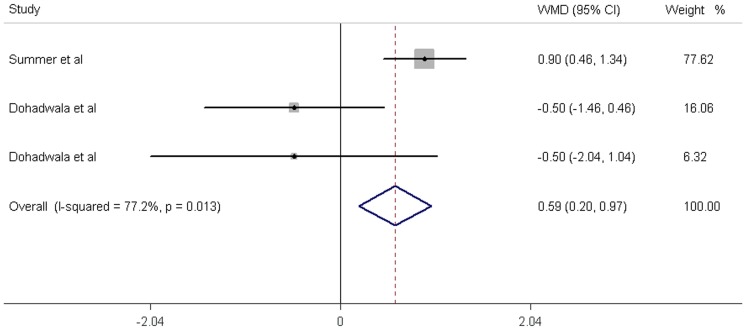
Meta-analysis of effects of fruit juice on HOMA-IR values. The result was obtained from a fixed-effects model. Sizes of data markers indicate the weight of each study in this analysis. WMD, weighted mean difference.

As shown in **Table 2** **,** fruit juice did not significantly affect the concentrations of fasting glucose, fasting insulin and HbA1c, while it significantly increased the HOMA-IR values. No significant heterogeneity was found in the concentrations of fasting glucose, fasting insulin and HbA1c. Significant heterogeneity was noted in the results of HOMA-IR (*P* = 0.01). For the 12 trials that reported data on fasting glucose concentration, no significant mean differences was found in the subjects supplemented with fruit juice (0.79 mg/dL; 95% CI: −1.44, 3.02 mg/dL; *P* = 0.49; **Figure 2**) compared with control subjects. The mean difference change in fasting insulin concentrations was reported in 5 trials and found to be not significantly different (−0.74 µIU/ml; 95% CI: −2.62, 1.14 mg/dL; *P* = 0.44; **Figure 3**). In addition, the results of the effects of fruit juice on HbA1c concentrations and HOMA-IR values were −0.03% (95% CI: −0.28, 0.23%; *P* = 0.84) and 0.59 (95% CI: 0.20, 0.97; *P*<0.01), respectively (**Figure 4** **and** **Figure 5** **)**.

**Table 2:** Pooled effects of fruit juice on fasting glucose, fasting insulin, HbA1c and HOMA-IR levels.

Variable	No. of studies	Sample size (treatment/control)	Net change (95% CI)	*P*	Test of heterogeneity	
					*P*	*I^2^,%*
Fasting glucose (mg/dL)	12	279/276	0.79 (−1.44, 3.02)	0.49	0.68	0.0
Fasting insulin (µIU/ml)	5	149/156	−0.74 (−2.62, 1.14)	0.44	0.48	0.0
HbA1c (%)	3	56/54	−0.03 (−0.28, 0.23)	0.84	0.20	38.5
HOMA-IR (units)	3	121/119	0.59 (0.20, 0.97)	<0.01	0.01	77.2

HbA1c, hemoglobin A1c; HOMA-IR, the homeostatic model assessment of insulin resistance.

### Subgroup and Sensitivity Analysis

Pre-specified subgroup analyses showed that the pooled effects of fruit juice on fasting glucose concentrations were not influenced by population region, baseline glucose concentration, duration, type of fruit juice, GI values of fruit juice, fruit juice nutrient constitution, total polyphenols dose and Jadad score. The consumption of fruit juice significantly increased fasting glucose concentrations in parallel-design groups. Results are presented in **Table 3**. Sensitivity analysis showed that the pooled effects of fruit juice on fasting glucose were not altered when analyses were limited to high-quality studies and were not changed after imputation using a correlation coefficient of 0.5. In addition, we found no significant change of outcome measures through systematic removal of each trial during sensitivity analysis.

**Table 3:** Subgroup analyses of fasting glucose stratified by previously defined study characteristics.

Variables	Fasting glucose				
	No. of trials	Net change(95% CI)	Test of heterogeneity		*P*
			*P*	*I^2^,%*	
Subgroup analysis					
Region					
US	9	0.95 (−1.15, 3.36)	0.43	0.5	0.44
European	2	−1.25 (−10.72, 8.22)	0.73	0.0	0.80
Asia	1	0.50 (−7.24, 8.24)	–	–	0.90
Baseline glucose concentration					
<110 mg/dL (normal glucose)	7	1.03 (−1.29, 3.42)	0.39	5.9	0.60
>110 mg/dL (hyperglycemia)	4	−2.19 (−10.46, 6.08)	0.95	0.0	0.39
Study design					
Parallel	8	3.48 (0.44, 6.53)	0.84	0.0	0.03
Crossover	4	−2.53 (−5.63, 0.94)	0.99	0.0	0.16
Duration					
<7 wk (low median)	6	0.92 (−2.88, 4.72)	0.94	0.0	0.64
≥7 wk (high median)	6	0.72 (−1.44, 3.02)	0.21	29.9	0.61
Type of fruit juice					
Berries juice	4	3.28 (−1.05, 7.61)	0.73	0.0	0.14
Grapes juice	3	−0.56 (−3.82, 2.69)	0.09	57.7	0.74
Pomegranate juice	3	1.77 (−4.39, 7.93)	0.98	0.0	0.57
Orange juice	1	−1.80 (−11.78, 8.18)	–	–	0.72
Glycemic index of fruit juice					
Low glycemic index (≤55)	10	0.24 (−2.19, 2.67)	0.75	0.0	0.85
Medium glycemic index (56–69)	2	3.71 (−1.90, 9.32)	0.28	14.0	0.20
Fruit juice nutrient constitution					
Multi-nutrients	6	1.73 (−2.00, 5.47)	0.98	0.0	0.36
Polyphenols (main nutrients)	6	0.27 (−2.51, 3.05)	0.20	31.0	0.85
Total polyphenols dose					
<933.6 mg/d (low median)	6	2.60 (−0.81, 6.01)	0.13	0.0	0.69
≥933.6 mg/d (high median)	3	−0.78 (−4.06, 2.50)	0.28	0.0	0.45
Jadad score					
Low (<4)	6	1.69 (−1.85, 5.23)	0.96	0.0	0.35
High (≥4)	6	0.20 (−2.67, 3.07)	0.23	27.8	0.89

### Publication Bias

**Figure 6:**
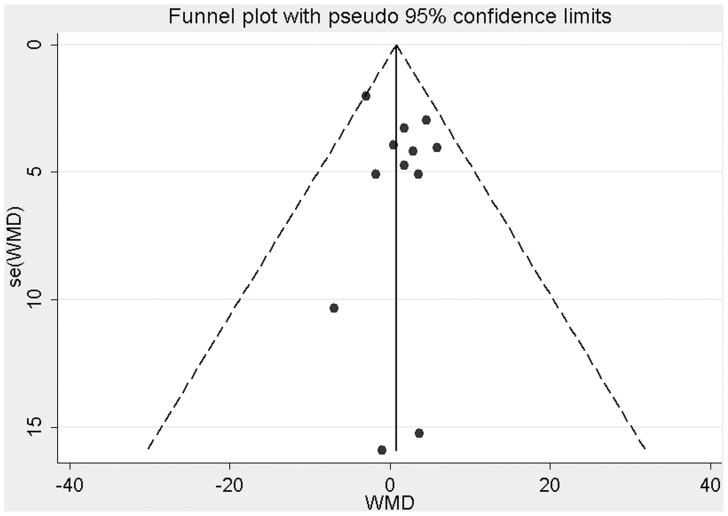
Funnel plots for the studies of the association of fruit juice consumption and fasting glucose concentrations.

The shape of funnel plot for the studies on fruit juice and fasting glucose did not show obvious publication bias (**Figure 6**). Similarly, no evidence of publication bias was observed by Egger’s test (*P* = 0.50). Publication bias of the studies on fasting insulin, HbA1c and HOMA-IR was not assessed owing to the limited numbers of studies currently available (n = 5, 3 and 3, respectively).

## Discussion

Our meta-analysis showed that fruit juice consumption did not significantly affect fasting glucose and insulin concentrations. Subgroup analyses further suggested that the pooled mean difference changes of fasting glucose concentrations were not significantly influenced by the population region, baseline glucose concentration, duration, type of fruit juice, GI values of fruit juice, fruit juice nutrient constitution and total polyphenols dose, and the outcome remained non-significant when analyses were limited to high quality studies. Our assessment of the effects of fruit juice on HbA1c and HOMA-IR values was limited by the small number of studies currently available.

As one of the most popular beverages, the global consumption of fruit juice has been steadily increased in recent years, probably due to the public perception of fruit juice as a natural source of nutrients [50]. It has been demonstrated that the polyphenols contained in fruit juice can improve the antioxidant status and immune function of the participants, thus may have beneficial effect in reducing the risk of cancer and cardiovascular disease [51], [52]. However, the role of fruit juice consumption on diabetes control has not been well studied and the result remains inconclusive. In this meta-analysis study, we found that fruit juice had no significant effects on fasting glucose and insulin concentrations. One possibility is that fruit juice has less fiber than whole fruit, and a previous meta-analysis indicated that increasing consumption of dietary fibers can reduce fasting glucose concentration and HbA1C [53]. Another possibility is that fruit juice intervention might modestly increase the participants’ dietary consumption of sugars and energy, which may influence the total effects of fruit juice on glucose control since most of the trials suggested that the participants maintained their usual diet during the intervention duration. In addition, previous studies suggested that fruit juice consumption had no significant favorable effect on lipid abnormalities, which often clusters with insulin resistance [54], [55]. Therefore, the effects of fruit juice on glycemic control in this study might be mildly underestimated, since the participants in the majority of the selected studies (10/12) had abnormal lipid profiles [38], [40]–[46], [48], [49].

We found that fruit juice intake significantly increased fasting glucose concentrations when we pooled the data of the parallel-design RCTs. However, this increasing effect might not be clinically significant since the participants in most selected parallel-design studies (6 of 8) had normal baseline glucose concentration, which can lead to a certain glucose fluctuation in the normal regulation of glucose homeostasis [56]. Therefore, we conducted an additional subgroup analysis using parallel-design trials which included participants with abnormal baseline glucose concentration, and the result suggested that fruit juice does not increase glucose concentration (*P* = 0.90). This result partly supported our inference, although it had less statistical power since only 2 trials were included in this subgroup analysis. In addition, we consistently found that fruit juice consumption had no significant effects on increasing glucose, when we conducted subgroup meta-analyses by the other variables, such as population region, baseline glucose concentration, duration, type of fruit juice, fruit juice nutrient constitution, total polyphenols dose or Jadad score. On the other hand, we also found that fruit juice could significantly increase the HOMA-IR values using the fixed effects model. However, the result is limited by the significant heterogeneity (*P* = 0.01) and the small number of available trials (n = 3). In addition, we found that fruit juice had no significant effects on HOMA-IR values when we used the random effects model (*P* = 0.87). For these reasons, whether fruit juice can affect the insulin sensitivity should be further evaluated.

It is suggested that the GI values indicate the extent of glycemic response to carbohydrate ingestion [57] and vary among different fruit juices [29]. To evaluate whether the GI values affects the association between fruit juice and glycemic control, we further investigated the effects of GI levels of fruit juices on fasting glucose concentrations. Our study indicated that neither low GI fruit juices nor medium GI fruit juices had significantly effect on fasting glucose concentrations. Since no selected RCTs used fruit juice with high GI as supplement, we could not evaluate the effect of high GI fruit juice on fasting glucose concentrations. In addition, this result was further limited because none of the selected 12 trials reported the GI values of the fruit juices they used. To minimize the difference between estimated and actual GI values, we indirectly obtained the most proximate GI values from the online GI database according to the types of respective fruit juices [29], and subsequently classified them in a certain range (low GI fruit juice, ≤55; medium GI fruit juice, 56–69) in the subgroup analysis. Consequently, more high-quality RCTs focused on the association between GI values of fruit juice and glycemic control are needed to further assess these causal conclusions.

To our knowledge, this is the first study to systematically review the potential effects of fruit juice on glycemic control. The relatively large amount of pooled participants possesses a greater statistical power than small number of subjects in a single RCT. However, some limitations should be addressed when interpreting the findings of this study.

At first, explicit doses of sugars, artificial flavoring agents and vitamins were unavailable in most of selected studies and the total polyphenols doses of fruit juice ranged from 341.9 to 2660 mg/d (median: 933.6 mg/d). Although the wide range of total-polyphenols dose did not result in the significant heterogeneity in our study, this might affect the overall outcomes of the meta-analysis.

Secondly, only 1 study excluded subjects with weight-reducing dietary regimen [41], and the remaining 11 did not report the information on the advice of losing weight. Ten of the 12 studies advocated that fruit juice was added to the usual diet, which might increase energy content of the diet and body weight of the participants. Nonetheless, 8 of the included studies reported non-significant changes in body weight and the remaining 4 studies did not report the related information. This potential discrepancy may partly due to the controlled amount of fruit juice suggested by the investigators (120–500 ml, or 50 g freeze-dried powders), which prevented the excess extra energy intake (150–350 kcal/d; information provided by 6 studies, [42]–[45], [48], [49]) of the participants. However, we could not further evaluate the association between fruit juice intake and body weight change owing to the limited available information on dietary structure, total energy intake and daily physical activities, etc. In addition, we could not conduct further subgroup analyses on the fasting insulin concentration, HbA1c and HOMA-IR due to the small number of available RCTs.

Thirdly, measures for glucose control or insulin sensitivity were not primary outcomes in some RCTs included in this meta-analysis and the null findings of secondary outcomes may not have always been published. In addition, although only randomized controlled trials were selected for our analysis which are inherently less susceptible to bias, the results synthesized from the 12 RCTs are still limited by their varied study qualities (from low to high), relatively small sample sizes (from 12 to 63) and short period of follow-up (from 4 wk to 3 mo). Moreover, only 2 studies used objective measures such as ellagic acid, or β-cryptoxanthin plus vitamin C to evaluate the intervention compliance [45], [48].

In conclusion, our study showed that the consumption of fruit juice may have no significant effect on fasting glucose and insulin concentrations. To further advance this area, future high-quality RCTs with adequate sample size and long follow-up periods are needed to evaluate and confirm the effect of fruit juice on glucose control and insulin sensitivity, particularly in the patients with hyperglycemia. To improve the quality of study, investigators should ensure and objectively evaluate the compliance, and use the appropriate blinding methods, control group and matched placebo. In addition, the result will be more convincing if they can effectively rule out the confounding effect of extra energy provided by fruit juice on body weight and other related measures.

## Supporting Information

Table S1 **Other characteristics of 12 randomized controlled trials included in analysis.**
(DOC)

Checklist S1 **Supporting PRISMA checklist.**
(PDF)
