# Supplementary material for: Effect of Fruit Juice on Glucose Control and Insulin Sensitivity in Adults: A Meta-Analysis of 12 Randomized Controlled Trials
Source: PLoS One. 2014 Apr 17;9(4):e95323. doi: 10.1371/journal.pone.0095323 (PMC3990696; doi:10.1371/journal.pone.0095323)
Supplement: Table S1 — Other characteristics of 12 randomized controlled trials included in analysis. (DOC) [file pone.0095323.s001.doc]

**Table S1.** Other characteristics of 12 randomized controlled trials included in analysis

| **Study** | **Control group** | **GI** | **Baseline glucose (mg/dL)** | **Body weight changes** | **Compliance surveillance** |
| --- | --- | --- | --- | --- | --- |
| Reshef 2005 | Placebo (low-flavonoid sweetie juice: 115 mg/d) The final HF and LF sweetie juice had similar contents of glucose, potassium, hesperidin (2.6 mg/L and 2.9 mg/L, respectively), and neohesperidin (4 mg/L and 3 mg/L, respectively). Both juices had similar texture, color, and taste. | 48 | 85.3 ± 9 | NR | NR |
| Summer 2005 | Placebo (modified sports beverage of similar caloric content, amount, flavor, and color) | 53 | 113 ± 30 | NS | NR |
| Bannni 2006 | No intervention | 52 | 90.4 ± 7.6 | NS | A 3-d diet record (consisting of 2 weekdays and a weekend day) was obtained from each subject. |
| Cerda 2006 | Placebo (synthetic orange flavoured drink lacking of antioxidants, fruit and vegetable extracts or vitamins) | 53 | 114.46 ± 25.53 | NR | Patients attended the consulting room at the hospital in the morning, the same day every week to answer physicians’ questions regarding the accomplishment of the protocol. |
| Hollis 2010 | Placebo (polyphenol-free substitute grape-flavored drink) | 52 | 78.48 ± 10.8 | NS | Participants were interviewed in person or by phone by a single interviewer using the University of Minnesota Nutrition Data System multipass software. |
| Basu 2010 | Water (similar fluid intake) | 53 | NR | NS | Participants were asked to return any unconsumed blueberry drink. For the 25 participants in the blueberry group, compliance was high, with 96.5% of the blueberry drink consumed and 100% compliance visits/wk. The controls reported 100% compliance in terms of water intake and biweekly visits. |
| Dohadwala 2010 | Placebo beverage (matched the flavor, color, calorie, and sugar profile of the juice, but did not contain any juice or polyphenolics) | 52 | 91 ± 10 | NS | Subjects were asked to return the juice container caps after each beverage consumption period, and the average compliance was 86%. |
| Basu 2010 | Water (similar fluid intake) | 40 | 93.6 ± 13.9 | NS | The researcher used ellagic acid as biomarker to evaluate compliance. Subjects were asked to bring back any unconsumed or left-over drink to assess unmonitored compliance. |

**Table S1. cont.**

| **Study** | **Control group** | **GI** | **Baseline glucose (mg/dL)** | **Body weight changes** | **Compliance surveillance** |
| --- | --- | --- | --- | --- | --- |
| Gonzalez-Ortiz 2011 | Placebo (NR) | 53 | 84.6 ± 5.4 | NS | NR |
| Dohadwala 2011 | Placebo beverage (no polyphenols) | 56 | 127 ± 49 | NR | The researchers assessed the compliance with the study protocol as the percentage of expected bottle caps that were returned. Average compliance was 94 6 8% for cranberry juice intervention group. |
| Morand 2011 | Control drink (only contains matched sugar composition) | 46 | 98.6 ± 15.0 | NS | Subjects kept a diary in which consumption of the study products (drinks and capsules) was recorded daily, and they were asked to return the unconsumed products at the end of each period. Compliance for the orange juice group was assessed by the plasma concentrations of β-cryptoxanthin and vitamin C. |
| Basu 2011 | Placebo (polyphenol-free cranberry-like taste drink matching glucose, vitamin C and energy) | 56 | 95.3 ± 7.3 | NR | All participants made 3 visits per week (Monday, Wednesday, and Friday) to ensure monitored compliance in cranberry juice or placebo consumption. Subjects were asked to bring back any unconsumed juice to assess unmonitored compliance. Compliance to juice and placebo intakes were 100% for the enrolled subjects. |

GI, glycemic index; NR, not reported; NS, non-significant.
